# Point-of-care detection of *Burkholderia pseudomallei*: A method integrating LAMP and LFSA with RNase HII hydrolysis

**DOI:** 10.1371/journal.pntd.0013109

**Published:** 2025-06-04

**Authors:** Yue Zhang, Xiuqiong Liang, Juan Yao, Wei Liu, Nan Zhang, Zhang Zhang, Qianfeng Xia

**Affiliations:** 1 NHC Key Laboratory of Tropical Disease Control, School of Tropical Medicine, Hainan Medical University, Haikou, Hainan, China; 2 Nanobiosensing and Microfluidic Point-of-Care Testing, Key Laboratory of Luzhou, Department of Clinical Laboratory, The Affiliated Traditional Chinese Medicine Hospital, Southwest Medical University, Luzhou, Sichuan, China; 3 Postdoctoral Research Workstation, Department of Neurosurgery, The First Affiliated Hospital of Hainan Medical University, Haikou, Hainan, China; Yale University School of Medicine, UNITED STATES OF AMERICA

## Abstract

Melioidosis, caused by *Burkholderia pseudomallei*, is a tropical disease known for its long incubation period and high mortality rate. However, the traits of this “great imitator” present significant challenges for clinical diagnosis and pose a serious threat to populations in epidemic regions. A rapid, accurate, and low environment requirement diagnostic method is needed to enable timely diagnoses. Here, we developed a strategy for point-of-care testing (POCT) using loop-mediated isothermal amplification (LAMP) with RNase HII for efficient detection of *B. pseudomallei*, through real-time fluorescence analysis or lateral flow strip assay (LFSA). Briefly, RNase HII-based cleavage dual-functional probe LAMP (RCDP-LAMP) utilizes RNase HII cleave the “*rG*” from the probe which hybridized with target. FAM group was cleaved, detaching from BHQ1, and exciting fluorescence or Biotin group on the same side as BHQ1 was used as the target captured in LFSA for the results. The limit of detection (LOD) for is 50 fg/reaction. In summary, RCDP-LAMP presents a versatile tool for detecting *B. pseudomallei* in both professional clinical laboratories and POCT scenes, suitable for large-scale screening efforts. This approach is essential for controlling costs in primary healthcare units and significantly enhances the prevention and management of the severely neglected tropical disease.

## Author summary

*Burkholderia pseudomallei* was first discovered in 1912 and is prevalent in tropical regions. It primarily persists in subsurface soil layers at 30 cm depth; extreme weather events such as tropical storms or sustained heavy precipitation can induce groundwater elevation and rainfall-driven soil erosion, mobilizing the pathogen to surface environments. Contact with the human respiratory tract and broken skin may lead to infection. *B. pseudomallei* can remain latent in the human body for months to years, with a rapid onset and high mortality rate. It also has natural resistance to a variety of first-line antimicrobial drugs. Timely, convenient, and easily accessible surveillance methods are crucial for diagnosis and treatment in resource-limited areas. We have proposed a detection method using Loop-Mediated Isothermal Amplification (LAMP) combined with RNase HII and a lateral flow strip, which meets the application conditions of the aforementioned regions. It is also suitable for screening during large-scale outbreaks, providing a new strategy and method for practitioners who are limited by various conditions and cannot identify *B. pseudomallei*. This method offers a timely, convenient, and easily deployable surveillance approach that is crucial for areas with limited resources.

## Introduction

*Burkholderia pseudomallei*, as an environmental Gram-negative bacillus, was reclassified into the novel genus in 1992 through 16S rRNA gene sequence homology and cellular characteristics [1]. Clinical cases of *B. pseudomallei* infection are atypical, presenting diverse symptoms that resemble those of other clinical diseases [2]. Limmathurotsakul’s model predicts that in 2015 alone, there were 165,000 cases of *B. pseudomallei* globally, resulting in 89,000 deaths [3]. Hainan, situated in a tropical region with a warm and humid climate, provides an ideal growth environment for melioidosis. From 2012 to 2021, a total of 825 cases of *B. pseudomallei* isolation were reported in Hainan, China [4]. Melioidosis is frequently underdiagnosed in many tropical nations that are endemic or potentially becoming endemic due to diagnostic limitations and lack of awareness [5]. Currently, no vaccine for c has been developed. It exhibits various drug resistance mechanisms collectively confer multidrug resistance to against multiple antibiotics. Due to its intracellular lifestyle, treatment requires prolonged intravenous infusions and oral antibiotic therapy. Without proper treatment, it may lead to recurrent infections [6–8].

The diagnostic capabilities for *B. pseudomallei* infection pose challenges, isolating and culturing from samples remains the gold standard in clinical diagnostics. However, this process requires a significant amount of time. Serological examination is a clinical method used to diagnose melioidosis. Nevertheless, the commonly used indirect hemagglutination assay (IHA) and enzyme-linked immunosorbent assay (ELISA) suffer from insufficient sensitivity [9–11]. In recent years, point-of-care testing (POCT) in immunology has emerged for diagnosing *B. pseudomallei*. In 2014, Houghton et al. described a diagnostic immunoassay for *B. pseudomallei* based capsular polysaccharide (CPS) detection using lateral flow immunoassay assays (LFIA), while Schully et al. developed a diagnostic kit targeting the CPS of *B. pseudomallei* [12,13]. Immunology based detection methods may not distinguish between past and asymptomatic infections, potentially leading to missed diagnoses when the pathogen is scarce in the body.

Molecular biology techniques offer greater sensitivity, specificity, and standardization for detection [14–17]. However, classical experiments have limitations. The high costs of laboratory construction and training professional personnel impose significant burden on grassroots experimental institutions [18]. Lengthy durations and low efficiency are major drawbacks, limiting their application in large scale or POCT screening [19]. Additionally, isothermal amplification methods require less stringent temperature control compared to PCR and have more flexible environmental and equipment demands. Examples include recombinase polymerase amplification (RPA), rolling circle amplification (RCA), crossing priming amplification (CPA), strand displacement amplification (SDA) and helicase dependent amplification (HDA) [20]. But complex procedures raise the barrier for operators, and the relatively insufficient specificity due to the reaction temperature being closer to room temperature. Notomi et al. established Loop-Mediated Isothermal Amplification (LAMP), characterized by rapidity, high efficiency, sensitivity, and specificity [21]. Furthermore, LAMP is 100–1000 times more efficient than PCR, which allows for earlier initiation and faster detection [22]. Recently, LAMP technology has been widely applied in identifying microorganisms across various industries, including clinical, marine, agricultural, and livestock, due to its high efficiency, sensitivity, and specificity in nucleic acid detection [23,24]. Chantratita et al. have applied LAMP technology for detecting melioidosis, demonstrating it as a viable alternative to PCR for rapid diagnosis of *B. pseudomallei* [25]. LAMP technology has undergone continuously improvements for detecting *B. pseudomallei.* Guo reduced detection time to under 20 minutes, while Tzeling introduced probes and an internal control into the detection system [26,27].

However, traditional LAMP technology urgently needs to address issues like false positives, target quantification, multiplex amplification difficulties, and challenges in amplifying short gene sequences [28]. Multiple primer pairs can cause nonspecific amplification of primer dimers, slowing the development of multiplex LAMP and leading to false positives in dye-based LAMP techniques [29]. JJoseph R. Dobosy et al. first applied RNase HII in RNase H dependent PCR (rhPCR), utilizing thermostable RNase HII for probe cleavage that breaks the DNA backbone at the 5’ side of a ribonucleotide sequence, yielding a 3’ hydroxyl group and a 5’ phosphate end [28,30,31]. Its unique recognition site provides a distinctive probe cleavage method, differing from TaqMan probes or the CRISPR/Cas system, significantly reducing false positives in LAMP that could lead to misdiagnoses [17,32]. To better utilize LAMP’s low instrumentation requirements, combining it with lateral flow strip assay (LFSA), which include an absorbent pad, interpretation area, and binding pad, is a worthwhile consideration. This method is flexible, simple, visually detectable, rapid, easy to operate, and easy to standardize, combining monoclonal antibody technology, chromatography, and colloidal gold particles. The combination of LFSA with RPA or PCR has proven to be a viable solution for detecting *B. pseudomallei* [33–35].

In this study, we utilize LAMP and LFSA technologies for rapid and straightforward detection of *B. pseudomallei* without complex instruments, making it suitable for POCT. Incorporating RNase HII significantly reduces the risk of false positives, enabling innovative strategies for preliminary *B. pseudomallei* screening in clinical and onsite testing, particularly in underserved regions. The RCDP-LAMP technique shows strong compatibility with reaction equipment, personnel, and laboratory facilities. Unlike traditional molecular diagnostic laboratories, its lower technological and equipment thresholds facilitate convenience for labs with basic infrastructure or field testing. Its quick and straightforward nature makes it ideal for large scale screening and POCT.

## Materials and methods

### Ethics statement

The use of clinical samples in this research institute has been approved by the Ethics Committee of Hainan Medical University (HYLL-2022-265), and the samples involved do not contain any specific information that can identify particular individuals.

### Materials and apparatus

Primers were synthesized and purified with Polyacrylamide Gel Electrophoresis (PAGE) method in this study by Tsingke Biotechnology Co., Ltd (Haikou, China). Probes were purified by high performance liquid chromatography (HPLC) Sangon Inc. (Shanghai, China). RNase-free ddH_2_O and dNTPs Mix were purchased from Sangon Inc. (Shanghai, China). The thermostable RNase-HII was acquired from Yeason Biotechnology Co., Ltd. (Shanghai, China). The Isothermal Amplification Buffer Pack (20 mM Tris-HCl, 10 mM (NH4)_2_SO_4_, 50 mM KCl, 2 mM MgSO_4_, and 0.1% Tween 20; pH 8.8 at 25 °C), Magnesium Sulfate (MgSO_4_) Solution and the Bst 2.0 DNA Polymerase were obtained from New England Biolabs (NEB, Ipswich, MA, USA). Nucleic acid amplification was conducted on Applied Biosystems QuantStudio 1 Plus (Waltham, MA, USA). Nucleic acid concentrations were measured with NanoPhotometer (ImplenGmbh, Munchen, Germania). The Clinx ChemiScope 6100(Shanghai, China) imaging platform was the tool for capturing the images. Nucleic acid lateral flow test strips were fabricated from Sangon Inc. (Shanghai, China) and Tianamp Bacteria DNA Kit was purchased from Tiangen Biotech (Beijing, China).

### Oligonucleotide design

To ensure the specificity of the detection *B. pseudomallei* by RCDP-LAMP, used NCBI Primer-BLAST website (www.ncbi.nlm.nih.gov) based on the whole genome (chromosome 1 and chromosome 2; GenBank accession numbers CP038805.1 and CP038806.1), designed Forward Inner Primer, Backward Inner Primer, Forward Outer Primer, Backward Outer Primer and RNase HII-base probe. To incorporated an RNase HII cleavage site into the probe that change the “G” at the eighth position to “*rG*”. The discriminating probe are modified at the 5’ end with a 6-carboxy fluorescein (FAM) group and 3’ end with a Biotin group which preserves the ability for subsequent binding of biotin to the LFA control line. Black hole quencher 1 (BHQ1) was modified in the middle of the probe, and all oligonucleotides used in this article are shown in Table 1. All oligos used in this study are shown in S1 Fig.

**Table 1:** Primers and probes used for this study.

Oligonucleotide	Sequence(5’to3’)	Length(nt)	%GC
**F3**	**GAGCGTACTAACGGGAATCG**	20	55
**B3**	**CCTCACTTCGAAGCCGAAC**	19	58
**FIP(F1c-F2)**	**GCACGGCGGAGATTCTCGAA CGTCGCTGGATGAGAAGAAA**	40	55
**BIP(B1c-B2)**	**AAGCCGCCTGCAAGTTTC GACGTGGGGGACGGGAG**	35	66
**Probe**	**5’-FAM/ACATCTC/*rG*/CTCT/ BHQ1/CCCAGGCC/3’Bio**	20	65

### Bacteria strains and genomic DNA extraction

*B. pseudomallei* isolated from a melioidosis patient, the whole genome sequencing has been completed and designated as HNBP001, in Hainan, China by our laboratory (GenBank accession numbers CP038805 and CP038806). The remaining clinical *B. pseudomallei* strains (No.1 to No.20) and the *Burkholderia cepacia* (*B. cepacia*) strains were isolated from clinical samples at the Second Affiliated Hospital of Hainan Medical University and confirmed by mass spectrometry. The strains used for cross-reactivity validation were obtained from American Type Culture Collection (ATCC). All bacteria strains were incubated at 37 °C with oxygen and cultured were shaken at 220 rpm in Luria Betani (LB) liquid medium. Genomic DNA extraction Used Tianamp Bacteria DNA Kit and followed the manufacturer’s instruction. Measuring the concentration of genomic DNA with NanoPhotometer.

### RCDP-LAMP assays

Each RCDP-LAMP assays system is 20 μL, comprises with 2 μL 10 × Isothermal Amplification Buffer, FIP and BIP (800 nM), F3 and B3 (200 nM), dNTPs (1.4 mM each), MgSO_4_ (5 mM), Probe (200 nM), RNase HII (0.5 mU/μL), Bst 2.0 DNA Polymerase (8 U/reaction) and Template (50 ng/reaction). The aforementioned components were added to the 0.2 mL PCR tubes on ice. The amplification procedure had been set to 60 °C for 60 minutes, while a shortened duration of 30 minutes was tested during optimization. Namely, 60 cycles, with fluorescence detected at the end of each cycle.

### Optimize the RCDP-LAMP assays

Optimize the RCDP-LAMP experiment mainly in terms of temperature, Mg^2+^ concentration, Bst 2.0 Polymerase concentration, the ratio of RNase HII and probe. With the same template amount of 50 ng per reaction, five temperature gradients are set: 55, 57.5, 60, 62.5, and 65 °C, and all other conditions being the same as in section RCDP-LAMP Assays. The optimal concentration exploration for Mg^2+^ is set between 2.5, 5, 7.5, and 10 mM. The gradient of Bst 2.0 DNA Polymerase is set at 4.8, 6.4, 8, 9.6, and 11.2 U/reaction. The ratio between the probe and RNase HII is dynamic. In the presence of a certain target gene, the same amount of probe may lead to insufficient cutting or waste of reagents under different concentrations of RNase HII, and vice versa. Probes at 200, 400, 600, and 800 nM concentrations and RNase HII at 10, 20, 40, and 80 mU/reaction are added to the optimization. Time to result (TTR) value being used to evaluation indicator. All experiments were performed in triplicate (n = 3).

### Sensitivity and specificity of RCDP-LAMP assays

The *B. pseudomallei* genome was diluted 5 ng, 500 pg, 50 pg, 5 pg, 500 fg, 50 fg and 5 fg per reaction for sensitivity. The templates were substituted by *E. coli, K. pneumonia, P. aeruginosa, S. maltophilia*, *S. aureus* and *B. cepacian* (50 ng/reaction) for specificity of RCDP-LAMP assays. Conduct experiments using the optimized reaction conditions. Among them, *S. maltophilia* as a differential diagnostic strain in clinical diagnosis and treatment of *B. pseudomallei*. It is worth noting that *B. pseudomallei* and *B. cepacia* both belong to the *Burkholderia* genus, and they also share certain similarities in isolation and identification. All experiments were performed in triplicate (n = 3).

### Clinical strains in the RCDP-LAMP assays

Clinical bacterial strains have been gathered from 20 melioidosis patients by our lab from Hainan’s local hospitals starting in 2019 for the examination of clinical samples (Table 2). The clinical strains were cultivated to log phase and extracted bacterial genomic DNA following the kit’s instructions. All experiments were performed in triplicate (n = 3).

**Table 2:** The bacteria strains in this study.

Strains	Origin	Sample categories	Strains	Origin	Sample categories
Bp No.1	Dongfang	Blood	Bp No.15	Wanning	Blood
Bp No.2	Dongfang	Bronchoalveolar lavage fluid	Bp No.16	Danzhou	Blood
Bp No.3	Ledong	Joint fluid	Bp No.17	Sanya	Bronchoalveolar lavage fluid
Bp No.4	Qiongzhong	Blood	Bp No.18	Changjiang	Blood
Bp No.5	Ledong	Blood	Bp No.19	Haikou	Blood
Bp No.6	Danzhou	Sputum	Bp No.20	Wenchang	Joint fluid
Bp No.7	Haikou	Sputum	Bc No.1	Wanning	Bronchoalveolar lavage fluid−
Bp No.8	Sanya	Urine	ATCC35218	−	*E. coli*
Bp No.9	Haikou	Blood	ATCC700603	−	*K. pneumonia*
Bp No.10	Dingan	Blood	ATCC27853	−	*P. aeruginosa*
Bp No.11	Changjian	Blood	ATCC17666	−	*S. maltophilia*
Bp No.12	Haikou	Blood	ATCC29213	−	*S. aureus*
Bp No.13	Dongfang	Blood	HNBP001	−	Isolated from our laboratory
Bp No.14	Dongfang	Bronchoalveolar lavage fluid			

### Lateral flow strip assay

The lateral flow strip was marked with anti-FAM antibody and anti-biotin antibody. The corresponding probes were cleaved by RNase HII into FAM part in 5’end and BHQ1 and biotin in 3’end. Post the RCDP-LAMP, amplification product was diluted to 60 μL of distilled water and fully mixed. Draw up 60 μL of the sample add to the absorbent pad, ensuring not to exceed the marked line. Results analyzed within 2–5 minutes later in the interpretation area, results obtained beyond the 5 minutes time frame shall be deemed inadmissible for interpretation. After the test strips had been sealed, they were disposed of safely. All lateral flow strips were single use and all experiments were performed in triplicate (n = 3).

### Data analysis

GraphPad Prism 9.5.0 (GraphPad Software, US) was used to import and analysis. Data were depicted in the form of histograms and subjected to analysis with a linear regression model. Twenty *B. pseudomallei* gene sequences isolated from clinical samples were tested using this method to assess its accuracy.

## Results

### Principle of RNase HII-base cleavage dual-functional probe LAMP (RCDP-LAMP)

**Fig 1:**
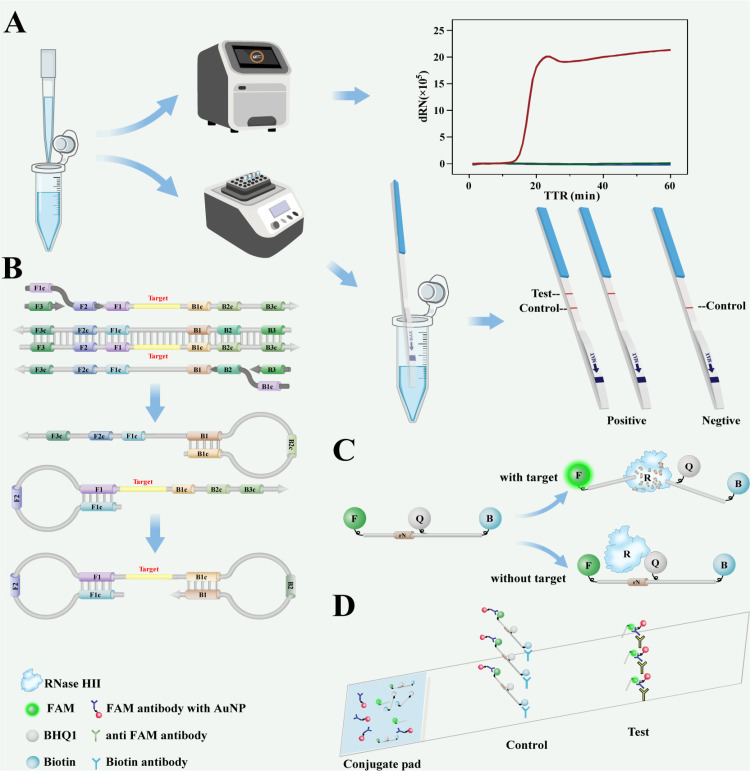
A schedule of RCDP-LAMP (A) From left to right. First, the reaction reagents are prepared and added to the PCR tubes. Next, use a real-time quantitative PCR instrument or any constant temperature device to control the reaction temperature. If conditions are limited, LFSA will be used instead. No template control (NTC) involves the addition of ddH_2_O instead of the target. (B) Schematic diagram of LAMP amplification in the reaction. FIP (F1c-F2) and BIP (B1c-B2) amplify, and then outer primers displace them. (C) The probe of experiment designed. When the target is bound with the probe, RNase HII will specifically recognize and cleave the *rN*, causing the FAM group to move away from the quencher and producing fluorescence. At the same time, the separation of Biotin from FAM can also meet the requirements of LFSA. (D) The operational principle of LFSA in action.

The principle of RNase HII-base cleavage dual-functional probe LAMP (RCDP-LAMP) on lateral flow strips as shown in Fig 1. Firstly, the sample is added to the reaction system for LAMP in a real-time quantitative PCR instrument or other isothermal devices. As shown in Fig 1B, the forward inner primer (FIP) and the backward inner primer (BIP) bind the target templates and amplify which the primers hybridize if the target is present in the RCDP-LAMP. Then, the forward outer primer (F3) and the backward outer primer (B3) hybridize to the target sequence, displacing the internal primer and its product, with the strand displacement reaction mediated by Bst2.0 polymerase. This forms an intermediate product of a single loop. Next, the inner primer on the opposite side binds to this product and extends, forming a dumbbell-shaped products. Based on the 2 pairs primer the specificity of amplification is guaranteed. To avoid the influence of primer dimers on the interpretation of results, a RNase HII-mediated signal output system was incorporated into LAMP (Fig 1C). At the same time, the specially designed probe has a FAM group inserted at the 5’ end and a Biotin group inserted at the 3’ end. A BHQ1 group is inserted between them, and G is replaced with “*rG*”. RNase-HII preferentially cleaves a dsDNA duplex containing a single ribonucleotide, leaving the FAM part separated from the BHQ1-Biotin part, leading to the generation of a fluorescent signal. This endows the probe with the function of monitoring signal changes in real time, and detection is carried out on the LFSA in a POCT manner (Fig 1D), can also be tested in the field.

### Optimization of experiment

**Fig 2:**
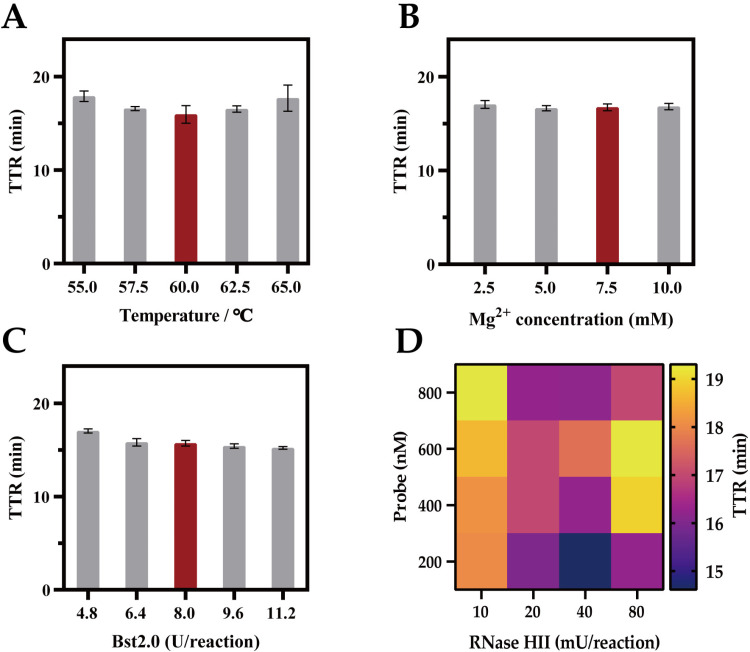
TTR values of RCDP-LAMP according to the different combinations. Temperature (A), Mg^2+^ concentration (B), Bst2.0 polymerase (C), probe and RNase HII (D). Error bars indicate ±1 SD from three independent replicates.

To optimize the reaction components’ concentrations, a series of tests were conducted on various temperatures, Mg^2+^ concentration, concentration of Bst2.0 polymerase, probe and RNase HII concentration. Using the genomic DNA (gDNA) template extracted from *B. pseudomallei* Hainan standard strain HNBP001. The results indicated the temperature of 60 °C was a better option in RCDP-LAMP (Fig 2A). The adjustment results for Mg^2+^ concentration showed little difference at 2.5 mM, 5 mM, 7.5 m and 10 mM, but a slight advantage was observed at 7.5 mM (Fig 2B). Although elevated concentrations of Bst2.0 polymerase progressively reduced the TTR, an enzyme dosage of 8 U per reaction was adopted in this study to balance reaction efficiency with cost-effectiveness (Fig 2C). The concentration and proportional relationship of probes and RNase HII were essential in the LAMP reaction. A suitable ratio would lead to an earlier TTR. a ratio of 40 mU RNase HII to 200 mM probe (1:5) was selected for each RCDP-LAMP reaction, ultimately (Fig 2D). Finally, the optimized conditions for the reaction are set for the RCDP-LAMP was 20 μL reaction volume comprises with 2 μL 10 × Isothermal Amplification Buffer, 800 nM FIP and BIP, 200 nM F3 and B3, 1.4 mM dNTPs, 7.5 mM MgSO_4_, 200 nM Probe, 40 mU RNase HII, 8 U Bst 2.0 DNA Polymerase for 60 °C.

### Evaluated sensitivity of RCDP-LAMP and lateral flow strip assay

**Fig 3:**
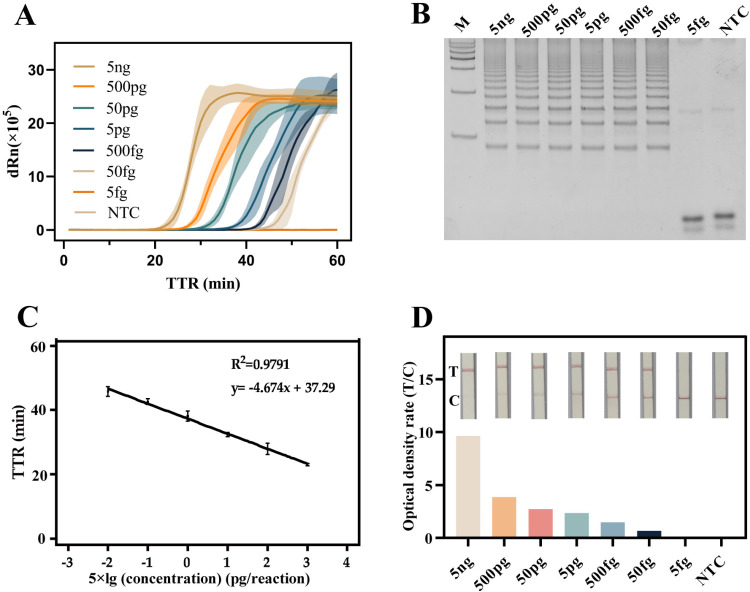
Sensitivity of RCDP-LAMP and LFSA. (A) Sensitivity of RCDP-LAMP real-time amplification curves with *B. pseudomallei* from 5 ng down to 5 fg per reaction and NTC. (B) Native PAGE gel with the sensitivity of RCDP-LAMP. The marker bands are arranged from top to bottom as follows 2000, 1500, 1000, 750, 500, 250 and 100 bp. (C) A stand curve with RCDP-LAMP was composed of TTR and 5 × lg concentration with pg/reaction. (D) LFSA of RCDP-LAMP with 5 ng to 5 fg per reaction and NTC after amplification. The bar chart shows the ratio of the optical density of T line to the C line. The NTC used ddH₂O as a substitute for the template. Results are representative of three replicates (n = 3).

The *B. pseudomallei* reference strain, HNBP001, were used for the post RCDP-LAMP optimization assessment. Generally, the sensitivity of a detection method is typically judged by the limit of detection (LOD), which represents the lowest concentration or smallest amount that the test method can detect. A lower LOD indicates a more sensitive detection method, in other words, it means patients can be diagnosed at an earlier stage of pathogen infection. The extracted gDNA of *B. pseudomallei* template was subjected to the RCDP-LAMP. As shown in Fig 3A, template of *B. pseudomallei* were diluted from 5 ng/μL to 5 fg/μL (5 ng, 500 pg, 50 pg, 500 fg, 50 fg, 5 fg per μL). The real-time fluorescence curves exhibited a sigmoidal pattern across gDNA template concentrations ranging from 5 ng to 50 fg per reaction. The products were analyzed using the test strips immediately when the RCDP-LAMP finished. The amplification curves show that positive results were obtained at concentrations of 5 ng, 500 pg, 50 pg, 5 pg, 500 fg and 50 fg per reaction, and the 5 fg/reaction was negative in 60 min. The native PAGE gel proved that the 5 fg/reaction was not detected (Fig 3B). All positive samples produced a ladder like typical LAMP result image in the PAGE gel. To identify the detectable samples and their corresponding TTR values on the coordinate axis, and a standard curve was created. Results are shown a negative linear correlation between them. The linear mathematical model is TTR = -4.674 × lg(C)+37.29(R² = 0.979) (Fig 3C). Amplification products were diluted and added to the test strip. The tests were conducted to ascertain the sensitivity threshold of the LFSA. The “Test” line would appear or both “Control” and “Test” lines would display simultaneously under the action of RNase HII. This indicated a positive result. Without target fragments being amplified, the probe would not be cleaved that all probes are bound by the anti-biotin antibody. Leading to only the “Control” line would appear. As shown in Fig 3D, the “Test” line for *B. pseudomallei* shows a positive result at concentrations ranging from 5 ng to 50 fg per reaction, similar to the RCDP-LAMP results.

### Specificity of RCDP-LAMP and lateral flow strip assay

**Fig 4:**
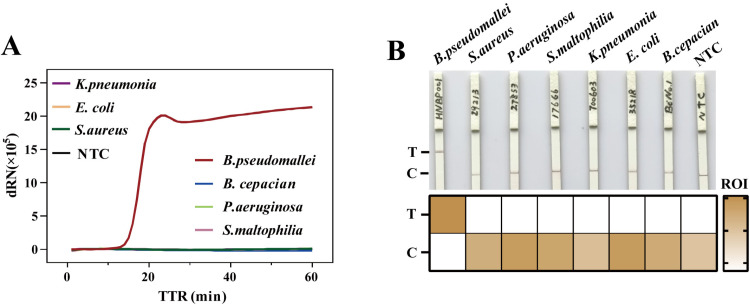
Specificity of RCDP-LAMP and LFSA. (A) Amplification curves of specificity of RCDP-LAMP among gram negative *E. coli* (ATCC35218), *K. pneumonia* (ATCC700603), *P.aeruginosa* (ATCC27853), *S. maltophilia* (ATCC17666), *S. aureus* (ATCC29213) and clinically isolated *B. cepacian* and *B. pseudomallei* (HNBP001). (B) The LFSA bands are from top to bottom as follows “Test” line and “Control” line. These test strips represent the experimental results of the amplified samples in A. The heatmap below the photo shows the absolute values of the optical density of the T and C lines. The NTC used ddH₂O as a substitute for the template. Results are representative of three replicates (n = 3).

Specificity refers to the proportion of actual non-diseased samples that are judged as negative by the diagnostic test. The amplification principle of LAMP provides an extraordinarily high positive rate, including false positives. This can also be attributed to the interaction of multiple primers, as well as amplification at lower temperatures. To ascertain the analytical specificity of the RCDP-LAMP method for the detection of *B. pseudomallei* before its practical use. The method based on RNase HII cleavage probes offers excellent specificity to RCDP-LAMP (Fig 4A). In the specificity experiment with a total of 8 samples, the amplification curve showed that only the *B. pseudomallei* gDNA was detected. These outcomes substantiate that the RCDP-LAMP test is highly specific and efficient for the accurate detection of *B. pseudomallei* in the extracted gDNA without cross-reactivity or amplification of non-target species. The RNase HII exerts its function in RCDP-LAMP, in the presence of a target sample, the probes are cleaved into two parts, which is displayed on the lateral flow test strips (Fig 4B). Only the lateral flow test strips for the LAMP product of HNBP001 showed the “T” line.

### Clinical bacterial strains of *B. pseudomallei* application of the RCDP-LAMP and lateral flow strip assay

**Fig 5:**
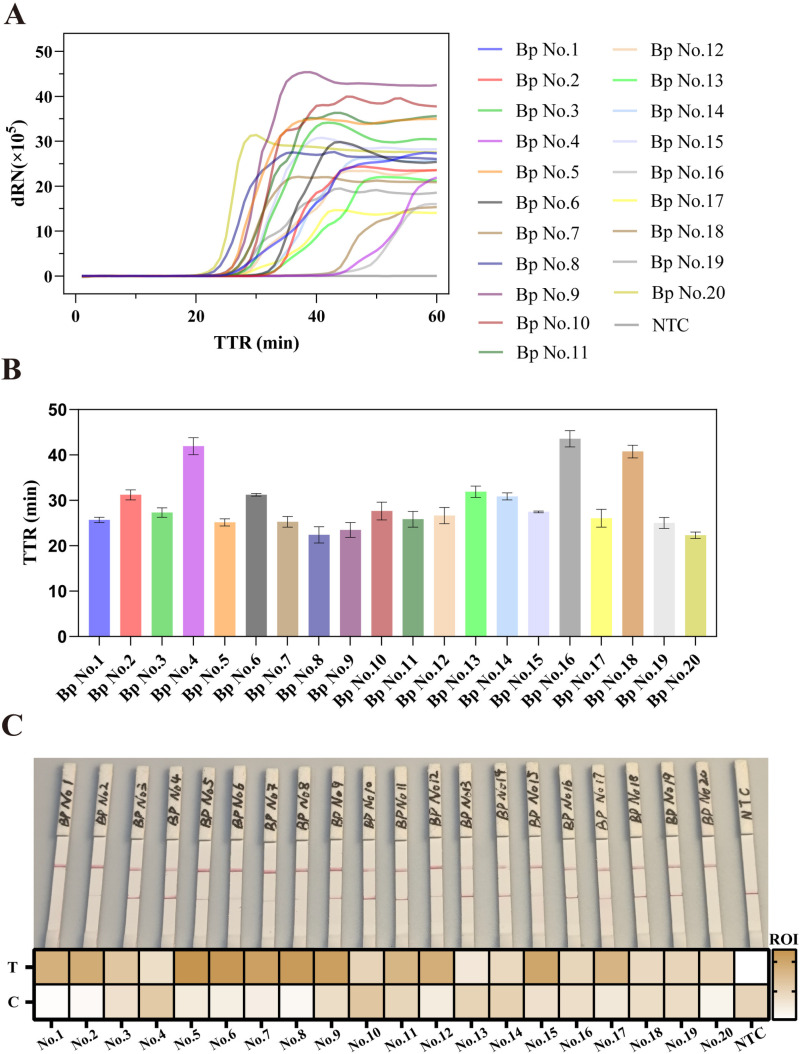
20 clinical samples test by RCDP-LAMP (A) Amplification curves of 20 clinical samples of B. pseudomallei and NTC are shown. (B) TTR values of 20 clinical *B. pseudomallei* samples. (C) LFSA result for the clinical samples are presented. The heatmap below the photo shows the absolute values of the optical density of the T and C lines. NTC involves the addition of ddH_2_O instead of the target into the reaction. Results are representative of three replicates (n = 3).

Twenty derived isolates from hospitalized patients’ cultures were used for the clinical application evaluation of RCDP-LAMP and LFSA. Results showed that all samples exhibited an upward trend in amplification curves, except for the NTC, which showed no amplification signal (Fig 5A). Even though the different genomic positions, sizes and template concentrations of the target genes result in distinct amplification curves, direct detection is more in line with the actual clinical application. Fig 5B displayed the TTR values for all samples. For the same reason, the results of the LFSA not completely match the standard results (Fig 5C). This includes the presence of the test line or both the control line and the test line.

## Discussion

*B. pseudomallei* became well known during the Vietnam War, as the tropical rainforests provide the most suitable environment for survival [2]. It causes melioidosis, which has led to post war soldiers suffering after a latency period of up to 60 years [7]. Due to the long incubation period, difficulty in diagnosis, and high mortality rate, melioidosis has been called a “neglected tropical disease” by the WHO [8]. This study proposes a detection platform POCT suitable, utilizing the principle of RNase HII specifically cleaving the probe when the target gene is present thereby releasing a fluorescent signal. Attributable to the explosive amplification of LAMP, the testing time of this reaction platform has been greatly reduced and the results are finally interpreted using LFSA. This method is highly adaptable for use in primary healthcare environments and can be significantly reduces the detection time of *B. pseudomallei*. Additionally, this platform is also compatible with real-time fluorescent detection. The target gene is interpreted based on the fluorescence curve takeoff. Consistent with LFSA, the platform uses fluorescence detection for result interpretation, achieving a LOD of 50 fg per reaction. Unlike the dye-based LAMP method, the addition of RNase HII significantly mitigates the risk of false positives.

In summary, the RCDP-LAMP study presents a detection platform that is fast, convenient, highly sensitive, and specific for *B. pseudomallei*. The integration of LFSA technology with LAMP offers lower costs, adaptability across testing environments, and is suitable for large-scale screening in economically underdeveloped regions. It promotes diagnosis, treatment, and control of *B. pseudomallei* in remote and underdeveloped areas, reduces the economic costs associated with *B. pseudomallei*, alleviates the burden on primary health care institutions in these areas, and provides new approaches for the prevention and control of *B. pseudomallei*. However, we acknowledge that RCDP-LAMP is not without flaws. Currently, most molecular diagnostic methods cannot directly use blood samples for pathogen detection, it also requires, and still requiring extraction of nucleic acids from the original samples [36]. The multitude of substances in blood affects the operation of PCR and other nucleic acid amplification methods. Overcoming this limitation could greatly advance in vitro diagnostics, facilitating quicker clinical diagnoses [37]. Additionally, the number of experimental samples also becomes one of the shortcomings of this platform. In the future, clinical samples that are collected will also be used for RCDP-LAMP. A larger sample size will further and more comprehensively improve the assessment of the platform’s detection effectiveness.

## Supporting information

S1 Fig Schematic of primers and probes. (A) Schematic of relative positions of nucleic acids; (B) and the genomic region of chromosome 2 in HNBP001 (2,484,151–2,484,571).
(TIF)
