# Supplementary figures and images for: Point-of-care detection of Burkholderia pseudomallei: A method integrating LAMP and LFSA with RNase HII hydrolysis
Source: PLoS Negl Trop Dis. 2025 Jun 4;19(6):e0013109. doi: 10.1371/journal.pntd.0013109 (PMC12136340; doi:10.1371/journal.pntd.0013109)

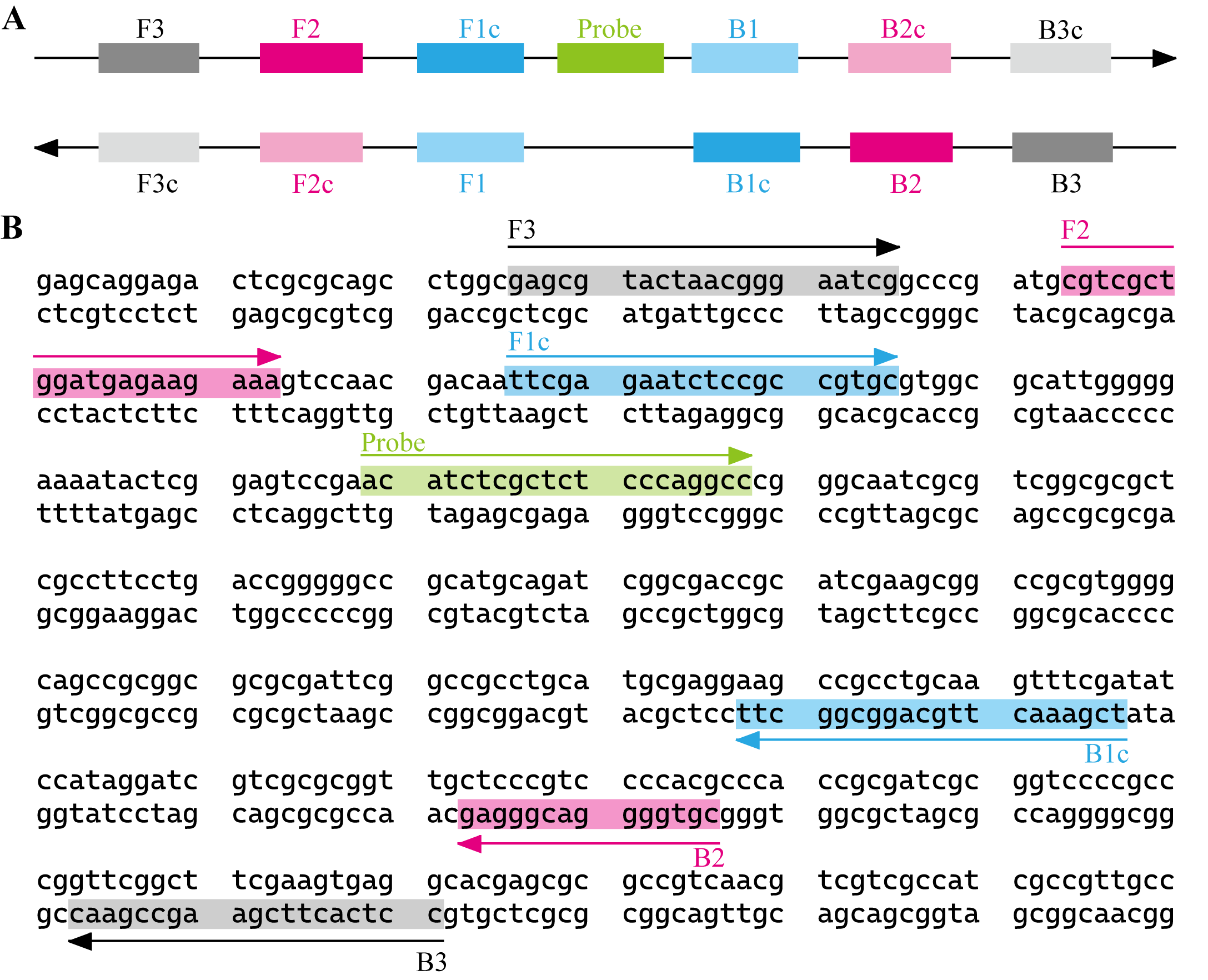

Supplement: S1 Fig — (A) Schematic of relative positions of nucleic acids; (B) and the genomic region of chromosome 2 in HNBP001 (2,484,151–2,484,571). (TIF) [file pntd.0013109.s001.tif]
